# Correction to Academic visualization study of aesthetic medicine management and related legal research since 2000

**DOI:** 10.1111/jocd.16736

**Published:** 2024-12-27

Deng K , Deng X , Luo H , et al. Academic visualization study of aesthetic medicine management and related legal research since 2000. J Cosmet Dermatol. 2024;23(8):2697–2710.38923263 10.1111/jocd.16327

In the author list, the affiliation number for Tianyu Li was incorrect. Tianyu Li should be associated with affiliation 4 (Department of Burn and Plastic Surgery, Nanshi Hospital Affiliated to Henan University, Nanyang City, Henan Province, China), not affiliation 2. Consequently, the corresponding address for Tianyu Li should be: Department of Burn and Plastic Surgery, Nanshi Hospital Affiliated to Henan University, Nanyang City, Henan Province, China.

We apologize for this error.

